# Comparison of two questionnaires to diagnose obstructive defecation syndrome during pregnancy and post-natally

**DOI:** 10.1007/s00192-022-05114-8

**Published:** 2022-03-10

**Authors:** Joanne Sentance, Katie Stocking, Richard J. Edmondson, Rohna Kearney

**Affiliations:** 1 Warrell Unit, Saint Mary’s Hospital, Manchester University NHS Foundation Trust, Oxford Road, Manchester, M13 9WL UK; 2 Faculty of Biology, Medicine and Health, School of Medical Sciences, The University of Manchester, Manchester, UK; 3 Centre for Biostatistics, Division of Population Health, Health Services Research and Primary Care, The University of Manchester, Manchester, UK; 4 Division of Cancer Sciences, Faculty of Biology, Medicine and Health, The University of Manchester, Saint Mary’s Hospital, Manchester, UK; 5 Manchester University NHS Foundation Trust, Oxford Road, Manchester, UK; 6 Division of Developmental Biology and Medicine, School of Medical Sciences, The University of Manchester, Manchester, UK

**Keywords:** Defecation, Pregnancy, Parturition, Puerperium, Anal sphincter, Surveys and questionnaires

## Abstract

**Introduction and hypothesis:**

Obstructive defecation syndrome (ODS) is a common urogynaecology presentation. This study compares two questionnaires, the electronic Personal Assessment Questionnaire (e-PAQ), used in urogynaecology clinics, with the ODS-Score (ODS-S), a simple validated scoring system used in colorectal clinics for diagnosing ODS, to identify patients with an ODS-S cut-off ≥9.

**Methods:**

A total of 221 paired ODS-S and e-PAQ questionnaires were completed; 80 during the second trimester of pregnancy, 73 during the third and 68 post-natally, including women sustaining obstetric anal sphincter injury (OASI). e-PAQ score and ODS-S were compared and Pearson’s correlation coefficient calculated. Areas under the curve assessed the diagnostic ability of e-PAQ scores to identify patients with ODS-S of ≥9.

**Results:**

The e-PAQ and ODS-S scores showed a positive correlation in the second and third trimesters of pregnancy, post-natally and following OASI. Pearson’s correlation coefficient was calculated (0.77; *p* < 0.001, 0.79; *p* < 0.001, 0.66; *p* = 0.001 and 0.79; *p* < 0.001 respectively). An e-PAQ evacuatory domain score of ≥33 identified women with an ODS score of ≥9 with a sensitivity and specificity of 71% and 94% in the second trimester, 86% and 95% third trimester and 78% and 97% in the OASI group respectively. Area under the curve was >0.90 for all groups.

**Conclusions:**

Comparison of e-PAQ evacuatory domain scores and ODS-S show a strong correlation, with an e-PAQ score of ≥33 promising for identifying women with an ODS score of ≥9, indicating ODS. This study will enable us to identify women during pregnancy and post-natally with ODS for whom early recognition and intervention may be beneficial.

## Introduction

Obstructive defecation syndrome (ODS) is a common presentation to urogynaecology and colorectal clinics. ODS is an evacuatory disorder characterised by a normal desire to defecate but an impaired ability to evacuate rectal contents [1] Symptoms include prolonged and difficult defecation, excessive straining, incomplete evacuation, pain, laxative use and requirement for digital manoeuvres [2, 3] Symptoms can be distressing and can significantly impact quality of life [1, 4–8]. Diagnosis for ODS is based on Rome IV criteria for functional constipation and defecation disorders and relies on symptoms, balloon expulsion test, anorectal manometry and/or anal surface electromyography (EMG). Although ODS is a common presentation, symptoms can be subjective, investigations invasive and of a sensitive nature, with varying treatment options and outcomes [9–12]. Self-reported questionnaires are increasingly used in the clinical setting to reliably assess symptoms, impact of disease and therapeutic outcomes [13]. Constipation is a prevalent symptom during pregnancy [14–16]. There are however limited data about the prevalence of obstructive defecation symptoms during pregnancy and post-natally, in particular following obstetric anal sphincter injury (OASI).

The ODS-Score (ODS-S) is a simple, validated five-item questionnaire designed specifically for the diagnosis of ODS, used commonly by colorectal surgeons but not currently used in urogynaecology clinics. A score of ≥9 suggests a diagnosis of ODS, with a sensitivity of 92% and specificity of 96% [10]. The electronic Personal Assessment Questionnaire (e-PAQ) is a validated, web-based clinical assessment tool widely used in urogynaecology clinics for the assessment of pelvic floor symptoms. The bowel evacuatory domain consists of seven questions, comparable with the ODS-S, with a possible score out of 100 (Table 1) [17].

**Table 1:** Obstructive Defecation Syndrome Score (*ODS-S*) and electronic Personal Assessment Questionnaire (*e-PAQ*) scoring systems for obstructive defecation syndrome (*ODS*)

Symptoms/variables	0	1	2	3	4
Five-item ODS-S (Renzi et al.) **[**10**]**					
Excessive straining	Never^a^	Rarely	Sometimes	Usually	Always
Incomplete rectal evacuation	Never	Rarely	Sometimes	Usually	Always
Use of enema/laxative	Never	Rarely	Sometimes	Usually	Always
Vaginal/perineal digital pressure	Never	Rarely	Sometimes	Usually	Always
Abdominal discomfort/pain	Never	Rarely	Sometimes	Usually	Always
e-PAQ evacuatory domain (Radley et al.) [17]					
Incomplete evacuation	Never	Occasionally	Most of the time	All of the time	–
Straining evacuation	Never	Occasionally	Most of the time	All of the time	–
Painful evacuation	Never	Occasionally	Most of the time	All of the time	–
Evacuation duration (min)	<5	5-10	10-20	>20	–
Perineal splinting	Never	Occasionally	Most of the time	All of the time	–
Anal digitation	Never	Occasionally	Most of the time	All of the time	–
Unable to evacuate	Never	Occasionally	Most of the time	All of the time	–

^a^Never, never; rarely, <1/month; sometimes, <1/week, >1/month; usually, <1/day; > 1/week; always, ≥ 1/day

This study is aimed at comparing the e-PAQ questionnaire evacuatory domain with the ODS-S in a population of pregnant and post-natal women to evaluate the performance of the e-PAQ for the diagnosis of ODS.

## Materials and methods

### Study population

After approval by Research Ethics Committee 3, West of Scotland (18-WS-0154 IRAS 245719) two cohorts of women were recruited as part of a prospective cohort study investigating evacuatory symptoms during pregnancy, after delivery and following OASI. Women were recruited from routine antenatal and postnatal perineal clinics at a large UK tertiary referral university teaching hospital with over 9,000 births annually. All women were aged over 18 years and able to provide informed consent. Two cohorts of women were recruited. The first cohort (group one) were nulliparous women, in the second and third trimesters of pregnancy and followed up to 1 year post-natally. The second cohort (group two) were women attending perineal clinic after sustaining OASI up to 12 months following delivery of their first child. Exclusion criteria included women with a previous second- or third-trimester loss, fetal abnormality, multiple pregnancy, history of bowel disease (not including IBS), bowel, perineal or vaginal surgery or a pre-existing neurological disorder.

### Questionnaires

All women were asked to complete two questionnaires evaluating bowel evacuatory symptoms; the ODS-S and the bowel domain of the e-PAQ (Table 1). The ODS-S is a validated questionnaire consisting of five evacuatory questions. Response scales are based on frequency of symptoms ranging from 0 (never) to 4 (always) with a possible score out of 20. A cut-off score of ≥9 is diagnostic for ODS. The bowel domain of e-PAQ consists of 33 questions across five domains to include IBS, constipation, evacuation, continence and quality of life. Response scales range from 0 (never) to 3 (all the time). The domain score is derived by adding the sum of the response scores for each item and multiplying by a factor of 4.726 to give a total possible score of 0–100. ODS is an evacuatory disorder; therefore, direct comparison of the ODS-S and the evacuatory domain of the e-PAQ was made.

### Study design and data collection

During routine clinical appointments eligible women were identified based on the inclusion criteria and referred to a member of the research team by their health care professional. The study was explained and a patient information sheet provided. Written consent was obtained and women were asked to complete the paper ODS-S and the bowel domain of the e-PAQ). Patients were able to opt whether they preferred to complete the e-PAQ electronically via a secure link or on a printed paper copy in the clinic. Demographic information was also collected, including age, BMI, ethnicity and gestation, using a standardised data collection form. Group one participants were asked to complete the two questionnaires on three separate occasions: second trimester, third trimester and again post-delivery (up to 12 months) to explore the prevalence of ODS during pregnancy. Group two completed the two questionnaires on one occasion when attending the perineal clinic as follow-up care after OASI.

### Statistical analysis

Statistical analysis was performed using Stata 14 (StataCorp. 2015; Stata Statistical Software: Release 14; StataCorp, College Station, TX, USA). A scatterplot using ODS-S and e-PAQ evacuation domain scores was created for the second and third trimester, post-natal and perineal groups to assess correlation. Pearson’s correlation coefficient was calculated for each group to evaluate the concurrent validity of the ODS-S and e-PAQ scores. An area under the ROC curve was performed for each group to assess the ability of the e-PAQ scores to correctly identify those women with ODS-S ≥9 and <9. Sensitivity and specificity were calculated for different cut-offs for the e-PAQ.

## Results

Paired questionnaires were completed by 143 women. Some women completed both questionnaires on more than one occasion in the second or third trimester and post-natally so that 221 paired e-PAQ scores and ODS-S were available for analysis (Table 2).

**Table 2:** Electronic Personal Assessment Questionnaires (*e-PAQ*) and Obstructive Defecation Syndrome Score (*ODS-S*) completed in the second and third trimester and post-natal groups

Time points of completed questionnaires	Number of women	Number of paired questionnaires
Second trimester only	15	15
Third trimester only	7	7
Post-natal only	6	6
Second trimester and third trimester	53	106
Second trimester and post-natal	2	4
Third trimester and post-natal	3	6
Second and third trimester and post-natal	10	30
Perineal clinic post-natal	47	47
Total	143	221

For women completing questionnaires antenatally (group one) the mean age was 29 (SD = 5.1, *n* = 80, range 18–41) and the mean BMI was 26.9 (SD = 60, *n* = 80, range 18–41). Ethnic groups were self-reported by study participants and ethnic categories were defined by gov.uk based on the Office for National Statistics 2011 Census of England and Wales [18]. In group one, 65 women were white, 16 were Asian or British Asian, 7 were Black, African, Caribbean or Black British, 6 were of mixed or multiple ethnic groups and 2 were of other ethnic groups. Eighty women in their second trimester completed both the e-PAQ and the ODS-S. The e-PAQ and ODS scores are shown in Table 3. The number of women with an ODS score of 9 or greater was 7 (8.8%). We obtained paired data from 73 women in their third trimester (Table 3). Seven women had an ODS-S of ≥9 was 7 (9.6%). Postnatally, 21 women had information for both the e-PAQ and the ODS-S (Table 3). Two women had an ODS-S of ≥9 (9.5%).

**Table 3:** Mean Electronic Personal Assessment Questionnaire (*e-PAQ*) scores and Obstructive Defecation Syndrome Scores (*ODS-S*) for group one and group two

Scores	Group one						Group two	
	Second trimester (*n* = 80)		Third trimester (*n* = 73)		Post-natal (*n* = 21)		Perineal group (*n* = 47)	
	Mean (SD)	Range	Mean (SD)	Range	Mean (SD)	Range	Mean (SD)	Range
e-PAQ Score	16.6 (12.6)	0–52	16.8 (12.4)	0–52	20.5 (11.9)	0–43	14.2 (13.6)	0–48
ODS Score	3.8 (3.2)	0–16	4.1 (3.2)	0–17	4.5 (3.5)	0–14	4.1 (3.9)	0–14

In group two, 47 women completed both the e-PAQ and the ODS (Table 3). The mean age was 31 (SD = 4.4, *n* = 47, range 20–38) and the mean BMI was 24 (SD = 3.4, *n* = 43, range 18–31). In group two, 30 women were white, 12 were Asian or British Asian, 3 were Black, African, Caribbean or Black British and 2 were of other ethnic groups. In group two, 9 women (19.2%) had an ODS score of ≥9.

**Fig. 1:**
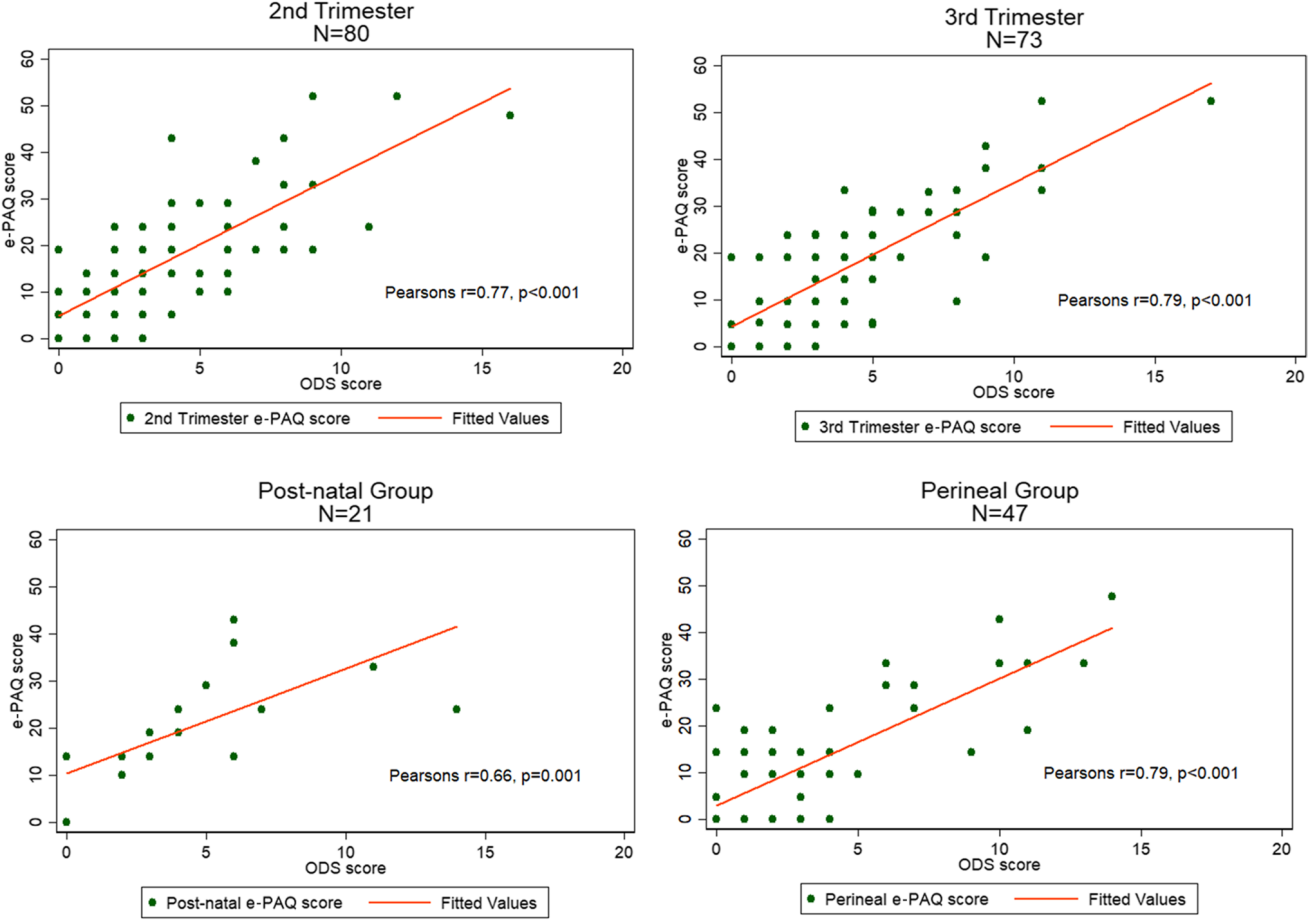
Scatterplots, Pearson’s correlation coefficient (r) and *p* values for the second- and third-trimester, post-natal and perineal groups for the e-PAQ evacuation domain scores

Scatterplots created to investigate the association between the e-PAQ bowel domain (0–100) and the ODS-S 0–20 showed a positive linear correlation for women in groups one and two. To assess the concurrent validity between the ODS-S and the e-PAQ scores, Pearson’s correlation coefficient was calculated. Scatterplots for the second- and third-trimester groups, the post-natal group and the perineal group are shown in Fig. 1.

**Fig. 2:**
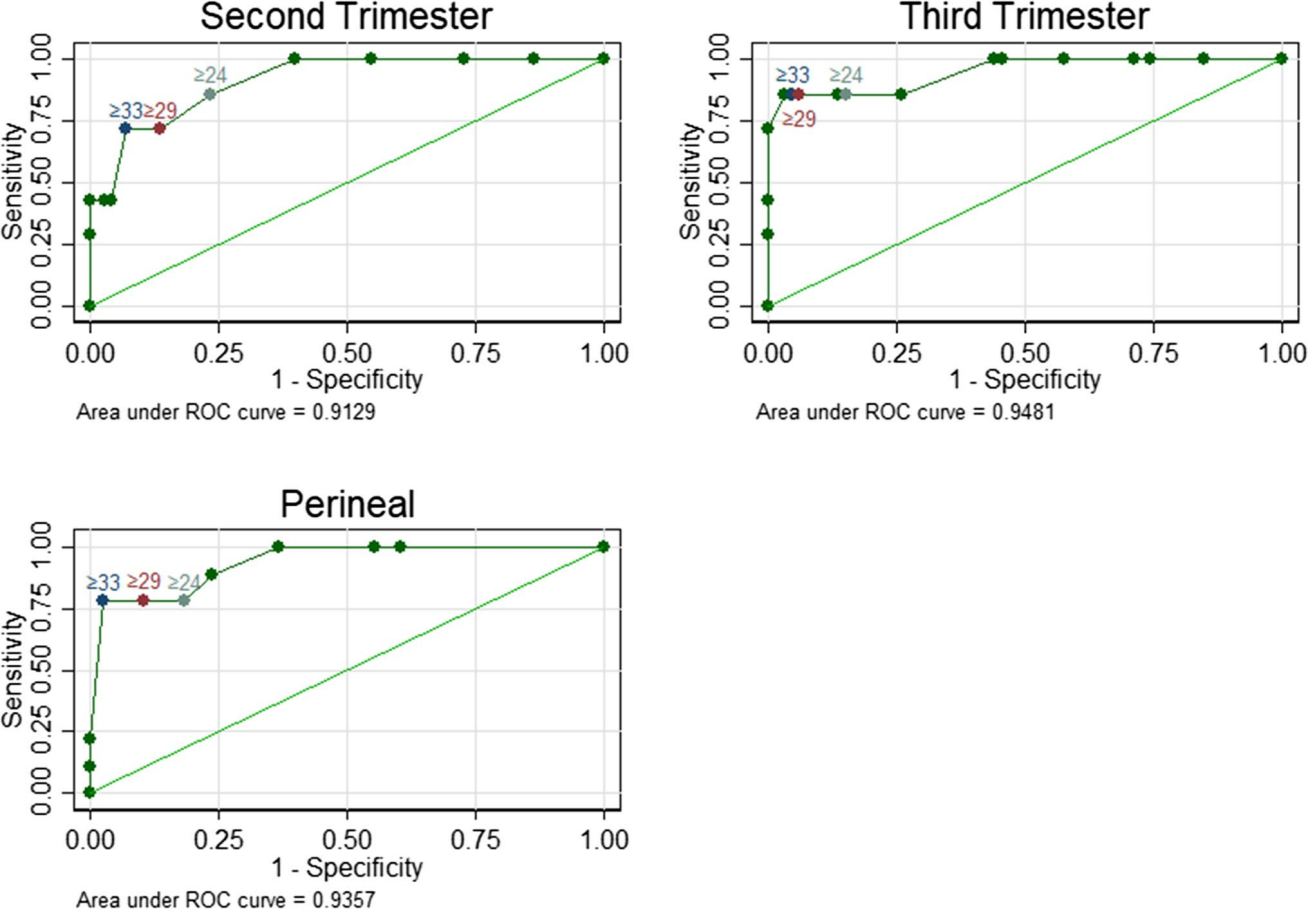
Receiver-operating characteristic (ROC) curves showing sensitivity and specificity for e-PAQ cut-off scores 24, 29 and 33 for the second-trimester, third-trimester and perineal groups

To identify an e-PAQ evacuation domain score that could reliably detect those women with ODSS ≥9, ROC curves were generated (Fig. 2) for each group and showed an area under the curve (AUC) of 0.91 for the second trimester, 0.95 for the third trimester and 0.94 for the perineal group. The group one post-natal group contained only two women with a score of ≥9 and so a ROC curve could not be calculated. Sensitivity and specificity were calculated for different e-PAQ cut-off points between 0 and 100 in the second and third trimesters and post-natally. e-PAQ evacuatory domain scores 24, 29 and 33 showed promising results, with a cut-off point score of ≥33, showing high sensitivity and specificity for identifying ODS-S < and ≥9 in all three groups (Table 4).

**Table 4:** Sensitivity and specificity values for electronic Personal Assessment Questionnaire (*e-PAQ*) evacuation domain scores 24, 29 and 33, in the second- and third-trimester and perineal post-natal groups

	Group one				Group two	
e-PAQ cut-off point	Second trimester		Third trimester		Perineal post-natal	
	Sensitivity (%)	Specificity (%)	Sensitivity (%)	Specificity (%)	Sensitivity (%)	Specificity (%)
≥24	85.7	76.7	85.7	74.2	77.8	81.6
≥29	71.4	86.3	85.7	93.9	77.8	89.5
≥33	71.4	93.2	85.7	95.5	77.8	97.4
AUROC	0.91		0.95		0.94	

*AUROC* area under the receiver operating characteristic curve

## Discussion

The aim of this study was to compare two validated questionnaires for the diagnosis of obstructive defecation syndrome during pregnancy and post-natally, including following OASI. The e-PAQ evacuatory domain was compared with the ODS-S to identify an e-PAQ score that correlates with an ODS-S ≥9, indicating ODS. The findings show a clear positive correlation between the two scoring systems with an e-PAQ score of ≥33 corresponding to an ODS-S ≥9 for the diagnosis of ODS with a sensitivity and specificity of 71% and 94% in the second trimester, 86% and 95% in the third trimester and 78% and 97% in the OASI group.

Constipation is a common symptom during pregnancy, thought to affect up to 40% of women at some point [14–16]. However, the incidence of constipation varies significantly within the literature, possibly because of differing definitions or lack of standardised methods of reporting [19]. Additionally, many of the studies conducted are retrospective, explore limited constipation symptoms and were undertaken more than 20 years ago [16].

Obstructive defecation syndrome refers specifically to evacuatory symptoms such as straining, incomplete evacuation, requirement for digitation, pain and use of laxatives, which are often distressing for patients and have a negative impact on quality of life [4–7]. These symptoms are commonly reported during pregnancy, but data are currently limited for prevalence and contributing factors [20, 21]. Van Brummen et al. investigated a range of bowel symptoms during pregnancy and post-natally using a non-validated questionnaire. Questions were selected, compared with the literature and international definitions, and three experts within the field were interviewed. Findings showed constipation rates of 8.9% at 12 weeks’ gestation; this reduced to 4.5% at 36 weeks’ gestation and remained stable at 3 and 12 months post-natally (4.6% and 4.2% respectively) [19, 21].

Derbyshire et al. used a bowel habit diary and compared this with the Rome II definition for functional constipation to report constipation, evacuatory and other gastrointestinal symptoms during pregnancy and post-natally. They found constipation rates to be highest in the first and second trimesters (35% and 39% respectively) falling to 21% in the third trimester and 17% post-natally [16, 19]. These studies provide some information about individual evacuatory symptoms during pregnancy and post-natally but are not validated for the diagnosis of ODS or routinely used in the clinical setting.

Several scoring systems for detecting constipation and ODS are discussed in the literature. The Patient Assessment of Constipation (PAC) is a paper-based validated questionnaire relating to bowel habits and is designed to assess the effectiveness of treatment for constipation but does not specifically detect symptoms of ODS [9, 10, 22]. The Cleveland Clinic Constipation Scoring System was designed as a symptom severity assessment tool; however, it was not prospectively validated and again includes non-specific symptoms for ODS [9, 10, 23]. Similarly the KESS scoring system, although prospectively validated and showing a positive correlation with the Cleveland Clinic Score, remains a scoring system that is primarily for constipation rather than specifically for ODS and has been found to ineffectively discriminate between patients with single or mixed pathological conditions [9, 24]. One scoring system designed specifically to assess the severity of ODS and response to treatment was developed by Altomare et al. [9]. This tool incorporates a clear definition of constipation and different subtypes and is prospectively validated. Although a promising ODS assessment tool, the questionnaire includes stool consistency, which is thought to vary significantly between patients over time and may be more representative of constipation, making it difficult to incorporate it in an ODS scoring system [10, 25].

The ODS-S selected for this study is a simple, prospectively validated tool, currently used by general surgical teams to assess for symptoms of obstructed defecation, with a clear cut-off score of ≥9 indicating disease. The ODS-S was developed from the Rome III criteria together with other internationally recognised questionnaires and is specifically for ODS [24, 26]. It can be used to diagnose ODS and evaluate the effects of interventions including surgery [10, 27]. The e-PAQ is a user-friendly, prospectively validated comprehensive pelvic floor questionnaire addressing urinary, bowel, prolapse and sexual symptoms and their effect on quality of life [17]. It is commonly available in UK hospitals and can be completed electronically in the clinic or on personal devices such as laptops, tablets or smart phones prior to appointments. To our knowledge, there is currently no research evaluating the use of the e-PAQ evacuatory domain for the detection and diagnosis of ODS.

This study shows a high correlation between the ODS-S and the evacuatory domain of the e-PAQ. By comparing the ODS-S, using the diagnostic cut-off score ≥9, with the evacuatory domain of the e-PAQ these findings have identified a corresponding e-PAQ score of ≥33 with high sensitivity and specificity for the diagnosis of ODS in the second and third trimesters and post-natally following OASI. By utilising the e-PAQ, a readily available, validated and robust scoring system for the assessment of ODS, this study allows early detection and management of ODS whilst providing further information about evacuatory symptoms during pregnancy and the post-natal period.

### Limitations

Women were recruited for this study in the antenatal and postnatal period with an age range of 18–41 years. Further work is required to validate the e-PAQ evacuatory domain for the diagnosis of ODS in non-pregnant and older populations. Primiparous participants were recruited to eliminate previous pelvic floor trauma as a contributing factor to ODS; therefore, validation in a multiparous population would provide further information about pregnancy and ODS. Owing to the current lack of data on ODS during pregnancy, a formal power calculation was not performed.

### Strengths

To our knowledge, there are currently no published data on the incidence of ODS during pregnancy and post-delivery. This study provides 221 paired questionnaires that show a clear positive correlation between the ODS-S and the e-PAQ evacuatory domain, allowing early recognition and treatment whilst offering further information about the prevalence of ODS during pregnancy and post-natally. The e-PAQ is a commonly available assessment tool and findings from this study form the basis for further investigation of ODS in wider populations for use in obstetric and gynaecology departments.

## Conclusion

Constipation is common during pregnancy, yet studies are dated, use varying definitions and often lack standardised reporting methods. There are currently very limited data on evacuatory symptoms during pregnancy and post-natally, in particular ODS. This study compares two validated questionnaires, the ODS-S with the e-PAQ evacuatory domain and shows a clear positive correlation, with an e-PAQ score ≥33 corresponding to an ODS-S ≥9 for the diagnosis of ODS.

The data provided by this study will enable health care professionals to utilise the e-PAQ to study women experiencing ODS during pregnancy and post-natally. Further research is required to validate the e-PAQ evacuatory domain for the diagnosis of ODS in non-pregnant and older populations.

## References

[1] Sharma S, Agarwal BB (2012). Scoring systems in evaluation of constipation and obstructed defecation syndrome (ODS). J Int Med Sci Acad.

[2] Arshad Rashid SK (2014). Obstructed defecation syndrome: a treatise on its functional variant. Intern Med.

[3] Bharucha AE, Wald A, Enck P, Rao S (2006). Functional anorectal disorders. Gastroenterology.

[4] Chou AB, Cohan JN, Varma MG (2015). Differences in symptom severity and quality of life in patients with obstructive defecation and colonic inertia. Dis Colon Rectum.

[5] Dennison C, Prasad M, Lloyd A, Bhattacharyya SK, Dhawan R, Coyne K (2005). The health-related quality of life and economic burden of constipation. Pharmacoeconomics.

[6] Irvine EJ, Ferrazzi S, Pare P, Thompson WG, Rance L (2002). Health-related quality of life in functional GI disorders: focus on constipation and resource utilization. Am J Gastroenterol.

[7] Belsey J, Greenfield S, Candy D, Geraint M (2010). Systematic review: impact of constipation on quality of life in adults and children. Aliment Pharmacol Ther.

[8] Dubois D, Gilet H, Viala-Danten M, Tack J (2010). Psychometric performance and clinical meaningfulness of the patient assessment of constipation—quality of life questionnaire in prucalopride (RESOLOR®) trials for chronic constipation. Neurogastroenterol Motil.

[9] Altomare DF, Spazzafumo L, Rinaldi M, Dodi G, Ghiselli R, Piloni V (2008). Set-up and statistical validation of a new scoring system for obstructed defaecation syndrome. Colorectal Dis.

[10] Renzi A, Brillantino A, di Sarno G, D’Aniello F (2013). Five-item score for obstructed defecation syndrome: study of validation. Surg Innov.

[11] Khaikin M, Wexner SD, Geibel J, Longo W (2006). Treatment strategies in obstructed defecation and fecal incontinence. World J Gastroenterol.

[12] Kamm MA, Nicholls RJDR (1997). Constipation. Surgery of the colon and rectum.

[13] Jenkinson C, McGee H (1998). Health status measurement: a brief but critical introduction.

[14] Anderson AS (1986). Dietary factors in the aetiology and treatment of constipation during pregnancy. BJOG.

[15] Jewell D, Young G, Kellie FJ. Interventions for treating constipation in pregnancy. Cochrane Database Syst Rev 2001;(2):CD001142. 10.1002/14651858.CD001142 PMC10799666 10796250

[16] Derbyshire EJ, Davies J, Detmar P (2007). Changes in bowel function: pregnancy and the puerperium. Dig Dis Sci.

[17] Radley SC, Jones GL, Tanguy EA, Stevens VG, Nelson C, Mathers NJ (2006). Computer interviewing in urogynaecology: concept, development and psychometric testing of an electronic pelvic floor assessment questionnaire in primary and secondary care. BJOG.

[18] UK Government, Ethnicity facts and figures, List of ethnic groups. Available at: https://www.ethnicity-facts-figures.service.gov.uk/style-guide/ethnic-groups. Accessed 8 Jan 2022.

[19] Shin GH, Toto EL, Schey R (2015). Pregnancy and postpartum bowel changes: constipation and fecal incontinence. Am J Gastroenterol.

[20] Brown S, Lumley J (1998). Maternal health after childbirth: results of an Australian population based survey. BJOG.

[21] Van Brummen HJ, Bruinse HW, van de Pol G, Heintz APM, van der Vaart CH (2006). Defecatory symptoms during and after the first pregnancy: prevalences and associated factors. Int Urogynecol J.

[22] Frank L, Kleinman L, Farup C, Taylor L, Miner P (1999). Psychometric validation of a constipation symptom assessment questionnaire. Scand J Gastroenterol.

[23] Agachan F, Chen T, Pfeifer J, Reissman P, Wexner SD (1996). A constipation scoring system to simplify evaluation and management of constipated patients. Dis Colon Rectum.

[24] Knowles CH, Eccersley J, Scott M, Walker SM, Reeves B, Lunniss PJ (2000). Linear discriminant analysis of symptoms in patients with chronic constipation: validation of a new scoring system (KESS). Dis Colon Rectum.

[25] Caetano AC, Dias S, Santa-Cruz A, Rolanda C (2018). Renzi score for obstructed defecation syndrome—validation of the Portuguese version according to the COSMIN checklist. Arq Gastroenterol.

[26] Renzi A, Izzo D, di Sarno G, Izzo G, di Martino N (2006). Stapled transanal rectal resection to treat obstructed defecation caused by rectal intussusception and rectocele. Int J Colorectal Dis.

[27] Williams AE, Croft J, Napp V, Corrigan N, Brown JM, Hulme C (2016). SaFaRI: sacral nerve stimulation versus the FENIX™ magnetic sphincter augmentation for adult faecal incontinence: a randomised investigation. Int J Colorectal Dis.

